# Inferring angiosperm phylogeny from EST data with widespread gene duplication

**DOI:** 10.1186/1471-2148-7-S1-S3

**Published:** 2007-02-08

**Authors:** Michael J Sanderson, Michelle M McMahon

**Affiliations:** 1 Department of Ecology and Evolutionary Biology, University of Arizona, Tucson, AZ 85721, USA; 2 Department of Plant Sciences, University of Arizona, Tucson, AZ 85721, USA

## Abstract

**Background:**

Most studies inferring species phylogenies use sequences from single copy genes or sets of orthologs culled from gene families. For taxa such as plants, with very high levels of gene duplication in their nuclear genomes, this has limited the exploitation of nuclear sequences for phylogenetic studies, such as those available in large EST libraries. One rarely used method of inference, gene tree parsimony, can infer species trees from gene families undergoing duplication and loss, but its performance has not been evaluated at a phylogenomic scale for EST data in plants.

**Results:**

A gene tree parsimony analysis based on EST data was undertaken for six angiosperm model species and *Pinus*, an outgroup. Although a large fraction of the tentative consensus sequences obtained from the TIGR database of ESTs was assembled into homologous clusters too small to be phylogenetically informative, some 557 clusters contained promising levels of information. Based on maximum likelihood estimates of the gene trees obtained from these clusters, gene tree parsimony correctly inferred the accepted species tree with strong statistical support. A slight variant of this species tree was obtained when maximum parsimony was used to infer the individual gene trees instead.

**Conclusion:**

Despite the complexity of the EST data and the relatively small fraction eventually used in inferring a species tree, the gene tree parsimony method performed well in the face of very high apparent rates of duplication.

## Background

Since the advent of efficient nucleotide sequencing technology in the 1980's, sampling of plant genomes to build species phylogenies has emphasized organellar markers, especially in the chloroplast genome, and a few nuclear loci such as ribosomal RNA genes. Though not universal (see e.g., [1-3]), phylogeneticists' avoidance of the nuclear genome of plants is in no small part due to its relative complexity – mainly the frequent occurrence of paralogous copies of genes derived from gene duplications [4]. Not only is polyploidy widespread in plants, but recent evidence derived from whole genome sequencing projects suggests a cryptic history of whole genome duplication and diploidization not predicted by cytogenetic evidence, including for example the prospect that *Arabidopsis *has undergone three complete genome doublings since the origin of seed plants, legumes two, and cereals two or more [5,6]. This contributes to already complex dynamics of gene family expansion and contraction driven by functional divergence [4]. In *Arabidopsis*, 65% of genes are members of gene families [7], and because of silencing of alternative paralogs in other taxa, in addition to sporadic background rates of gene duplication, phylogenetic studies will undoubtedly sample even more duplications as they increase in taxonomic scope.

Phylogenetic methods are relatively poorly adapted to inferring species trees from gene trees that contain duplications, despite steady work since Goodman et al.'s [8] pioneering paper [9-14]. Complicating matters further, homologous recombination in gene families (e.g. gene conversion) can add reticulate patterns to gene family histories. Most efforts to use nuclear markers have therefore focused on finding true single copy loci or on extracting subsets of orthologs from gene families [15-20]. However, the problem of detecting and extracting orthologs is itself quite challenging: a diversity of techniques have been proposed, ranging from reciprocal best BLAST searches to more phylogenetically driven approaches [3,21-26].

Thus plant biologists are now in the curious position of having increasingly rich and deep phylogenomic data sets but lack a full spectrum of tools to build species phylogenies from them. In addition to whole genome projects, large EST libraries have been assembled for dozens of crops and model plants. At the moment these data provide the most taxonomically broad source of potential phylogenomic data in plants, but they are characterized by numerous gene families and to date only orthologous subsets have been exploited to build species phylogenies [3,27,28]. The data themselves also present numerous challenges because of the laboratory methods by which they are extracted [29], the complex informatics procedures by which they are assembled [28,30], and the diversity of molecular variation at the level of expression that they reflect (e.g. alternative splice variants; [29]). This paper examines both the phylogenetic informativeness of the EST data and current methodologies for building species phylogenies from duplication-rich gene families to address the potential utility of such data for constructing the phylogeny of plants.

**Figure 1:**
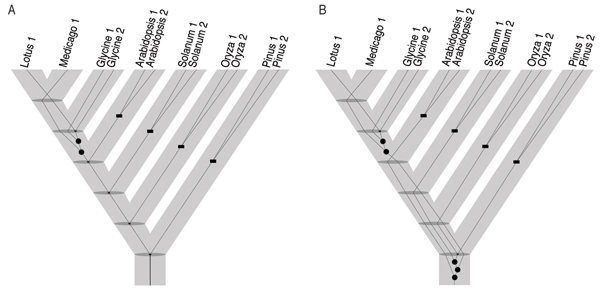
**Tree reconciliation example**. Two alternative rootings of the same unrooted gene tree (thin black lines) imbedded in a species tree (thick grey lines) visualized with the tool PrIMETV [76]. The gene tree is the maximum likelihood tree for a data set with 12 tentative consensus (TC) sequences assembled from ESTs from seven taxa (our cluster 13024). Bars indicate duplications within species (in-duplications) and black circles indicate out-duplications (those followed by a speciation event). A. The gene tree rooted to minimize the number of duplications required to reconcile the trees (two out-duplications required). B. The gene tree rooted using midpoint rooting, which places the root along the branch to the *Arabidopsis *sequences. This rooting is less optimal, requiring five out-duplications.

That the signature of species phylogeny can be found in complex gene trees displaying a mosaic of orthologous and paralogous relationships has been recognized for decades [31]. The first piece of possible strategy to infer such relationships was provided by Goodman et al. [8], who developed an algorithm for fitting a given species tree and gene tree together to determine the minimum number of duplications necessary to explain the data. This problem came to be known as "tree reconciliation", and several algorithms were developed to solve it efficiently [9,14,32]. Figure 1 illustrates some of the complexities involved. For example, a simple re-rooting of the gene tree can have dramatic effects on inferences about the history of gene duplication. The second element of the strategy is a search among candidate species trees, determining the minimum duplication score for each species tree relative to one or more gene trees that are assumed to be known [9,10,12,33-37]. This is an optimization problem entirely analogous to maximum parsimony or likelihood, but in which the optimality criterion is the summed duplication score (or perhaps the summed duplication plus loss scores) across all gene trees for a given species tree. The rationale for this "gene tree parsimony" (GTP) approach is that we should seek the species tree that imposes the fewest assumptions of unnecessary duplications in the collection of gene trees available. Though rarely used [33,36,37], Cotton and Page [12] showed in an extensive analysis of vertebrate gene families that it was possible to reconstruct a very credible species tree of vertebrates using this approach. One reason it has not been explored much in real data may be the lack of available software tools to implement the tree search part of GTP. Though several tools are available to do tree reconciliation [38-40], Page's program GeneTree [39], is the only widely available software to implement tree search heuristics, but these are relatively simple, having only tree rearrangement heuristics and no sequential addition steps.

**Figure 2:**
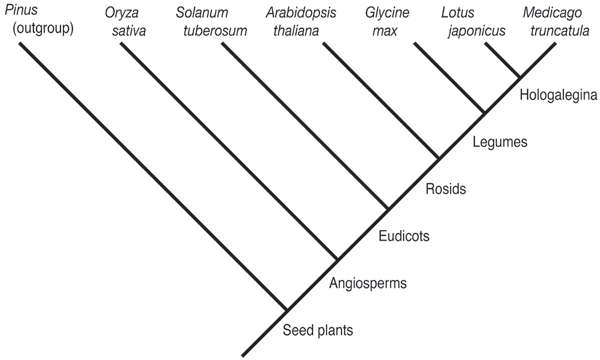
**Accepted species tree for seven plant model species**. Names of clades are indicated at internal nodes. See text for discussion of strength of evidence for this phylogeny.

One way to assess the utility of a phylogenetic method is to compare its output to a "known phylogeny". In this paper we examine the efficacy of GTP for reconstructing species relationships across angiosperms using an "accepted" angiosperm tree for six taxa, together with pine as an outgroup (Fig. 2). Limiting the problem to this size accomplishes two things: first, it permits exhaustive searches of the species tree space, avoiding the problem of developing efficient heuristics for searching tree space; second, it provides an immediate test of the quality of the results. The six angiosperms chosen span deep and relatively shallow phylogenetic relationships, ranging from the monocot-eudicot split (~120 Ma) to splits within one clade of eudicots, the legumes, which is a relatively recent radiation (~60 Ma). Phylogenetic relationships of these six angiosperms are strongly supported by numerous studies from multiple single copy (or effectively single copy in the case of 18S rDNA) loci [41-43], and in some cases from nuclear gene family data in which ortholog groups have been extracted [44]. In the case of legumes, the number of loci is fewer but both the monophyly of legumes and the indicated three-taxon statement within legumes are supported by multiple loci [45-47].

A final ingredient in any assessment of utility of methods and data is a statistical estimate of reliability of results. Agreement or disagreement of results with the accepted phylogeny takes on added meaning if the estimated tree is strongly supported. Little work has addressed confidence limits in gene tree parsimony studies. However, bootstrap procedures may provide some useful indication of strength of evidence [12]. In addition to the "usual" error expected in phylogenetics – incorrect gene trees owing to noise in the sequence data or bias in the inference – there is an additional important source of error stemming from incorrect rooting of the gene tree. Gene tree parsimony methods require that both the species and gene tree be rooted. Whereas rooting is generally accomplished in species-level phylogenetics by outgroup analysis (often after an unrooted analysis is completed), this is usually more problematic in gene families, because of the difficulty of identifying the correct ortholog for the entire ingroup. As suggested by several authors [13,32] one way to sidestep this source of error is to implement GTP across all rootings of each gene tree; in other words, to calculate the GTP score by finding the rooting that minimizes it for each gene tree. This conservative approach is adopted here.

Some terminology associated with gene family data warrants definition. We refer to gene duplication events as *in-duplications *(i.e. producing *inparalogs*, [26]) or *out-duplications *(producing *outparalogs*, Fig. 1). In-duplications result in descendants within a single species and are therefore inferred to have occurred since the most recent common ancestor of the species and its sister group. This can include within-species duplications (or species-specific alleles), or duplications that appear to be within-species because of incomplete species sampling. Because the descendants of an in-duplication remain in a single species, they cannot prefer one species tree to another. Out-duplications, in contrast, occur earlier than the most recent speciation event and produce descendants in two (or more) species. An out-duplication therefore can (indeed, must) disagree with the species tree and can contribute to the preference of one species tree over another.

## Results

### Sequence data and gene trees

The TIGR Gene Indices Database provided 172,900 Tentative Consensus (TCs) sequences for the seven focal taxa (Table 1). After discarding sequences for which there was no open reading frame (ORF) at least 500 nucleotides (nt) in length, 105,453 TCs remained. These were trimmed to their longest ORF, producing sequences with average length of 1094 nt (336 nt shorter, on average, than the original TCs).

Clustering of sequences implemented with BLAST and single-linkage clustering produced a wide diversity of cluster sets (Table 2) depending on how we set the minimum hit fraction, which is the set union of the sets of locally aligned sites (hits) reported by BLAST. With this minimum value set to zero, nearly 40,000 clusters were assembled, some 4423 of them phylogenetically informative. However, the largest contained 6565 sequences, and the sequences in it were extremely heterogeneous in length, sequence, and annotation, and were not homologous to any level that would be useful in phylogenetic inference. Clearly the stringency of overlap set by the minimum hit fraction was too low. When we increased the minimum hit fraction the size of the largest cluster was reduced and the data became more fragmented, as reflected in the increasing number of clusters (Table 2), but also more homogeneous within clusters. Ultimately we selected a hit fraction of 0.7 in an effort to maximize the amount of information retained while attempting to minimize within-cluster heterogeneity (see also e.g. Schlueter et al. [48]; who impose analogous requirements, although on fractional overlap of an entire hit rather than the set union of hits, as we do).

The chosen cluster set contained 88,864 clusters, only 577 of which were potentially phylogenetically informative; that is, they had at least four sequences and at least three taxa (most contained just a single sequence or a single taxon: Table 3 and Table 4). Fifty-nine clusters contained sequences from all seven taxa. The largest informative cluster contained 94 sequences, including several from each of the seven taxa. On the other hand, an extraordinarily large number of clusters, 79,122, were singletons (only one sequence). The contributions of each taxon to the final data sets ranged widely: from 315 to 1065 TCs and membership in 159 to 538 clusters (Table 1). The number of sequences excluded due to insufficiently long ORFs also varied tremendously across taxa (Table 1). In the end only 4536 TC sequences of the original 105,453 found their way into phylogenetic analysis (4.3%). This was about one tenth of the sequences produced by the least stringent clustering requiring 0% hit fraction overlap, but those clusters were largely unusable because of their heterogeneity as described above.

The collection of gene trees reconstructed using parsimony (henceforth "parsimony gene trees") was quite similar to that reconstructed under likelihood ("likelihood gene trees"). In fact, 354 of the clusters produced the same tree topology or same set of equally optimal tree topologies. In 34 other clusters the set of ML trees was a proper subset of the set of MP trees, and in one cluster the reverse was true. Finally, in 187 clusters, the set of MP trees and the set of ML trees were disjoint. Not surprisingly, these tended to be the clusters with more sequences (mean 12.4 sequences, whereas the mean across all 577 clusters was 7.9 sequences).

### Tree reconciliation: duplication scores on the accepted species tree

The distribution of the number of clusters inferred to have a given number of duplications is highly skewed for both parsimony and likelihood gene trees with many clusters having zero duplications but the maximum number of duplications in any cluster still being quite large (Table 5).

On the accepted species tree, the inference method affected the number of duplications inferred for 154 clusters but had no effect on the other 323 clusters. Of those for which it made a difference, in 87 clusters the likelihood gene trees fit the accepted species tree better i.e. with fewer duplications, and in 67 clusters the parsimony trees fit better.

### Gene tree parsimony: finding the optimal species tree

**Figure 3:**
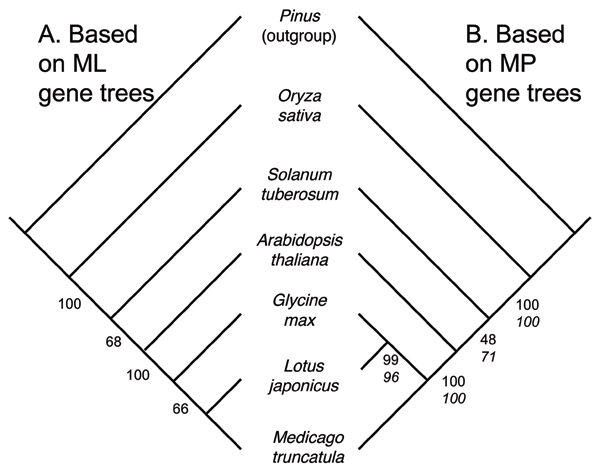
**Species tree inferred by gene tree parsimony**. A. The best species tree obtained using gene tree parsimony based on the maximum likelihood gene tree collection. It is identical to the accepted tree in Figure 2. B. The best species tree obtained using GTP based on the maximum parsimony gene tree collection. It differs from the accepted tree only within the legumes. Bootstrap II support values (resampling the gene trees: see text) are shown in plain text for each bipartition in the tree. Bootstrap I values (resampling the data within the original clusters) are shown in italics for tree B.

The optimal species trees differed slightly depending on whether the parsimony or likelihood gene trees were used. Based on the likelihood gene trees, the optimal tree was exactly the accepted species tree, with an (out-) duplication score of 779.0 (Fig. 3A). Based on the parsimony gene trees, the optimal species tree was very similar to the accepted tree, except for a rearrangement within the legumes (Fig. 3B). Its score was 771.9 (out-) duplications. The duplication score of the accepted species tree based on the parsimony gene trees was 796.3 and it was ranked fourth among all species trees. Fractional scores reflect weighting of multiple equally parsimonious or equally likely gene trees within a cluster. Note also that the rankings and relative scores are the same when counting all duplications as when counting out-duplications only. As they are restricted to only a single species, in-duplications are akin to autapomorphies in being phylogenetically uninformative.

**Figure 4:**
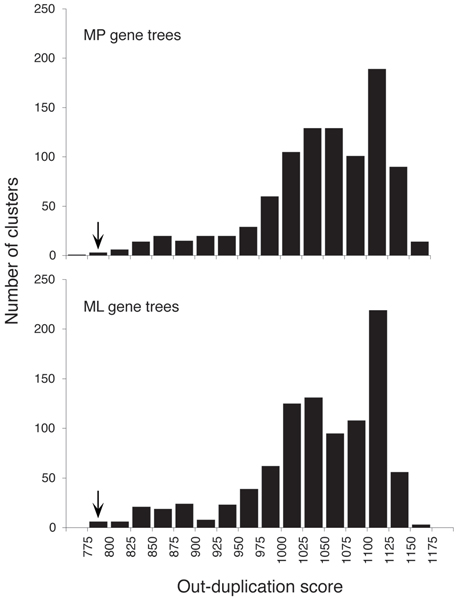
**Distribution of duplication scores across all species trees**. Distributions of out-duplication scores across all 945 binary angiosperm species trees (all rooted with *Pinus*). An out-duplication score is the sum of all out-duplications required to reconcile all 577 gene trees (or sets of trees) to that species tree. The upper panel shows the distribution of scores when the gene trees were estimated using maximum parsimony; the lower panel gives the same for the maximum likelihood gene trees. Arrows indicate the bins in which the accepted species tree occurs. For the MP gene trees, the accepted species tree was fourth from the best and had a score of 796.3 (the optimal species tree had a score of 771.9). For the ML gene trees, the optimal tree was the same as the accepted tree and had a score of 779.0.

Because of the exhaustive enumeration algorithm we could obtain the entire distribution of duplication scores for the two analyses (Fig. 4). The duplication scores for the likelihood trees ranged from 779.0 – 1152.0, whereas those for the parsimony trees ranged from 771.9 – 1165.9. Both distributions were highly skewed with a long tail of low scoring trees, suggesting the presence of phylogenetic signal, at least by analogy to skewness indices that have been used to study parsimony score distributions [49].

### Support levels and hypothesis testing

Bootstrap I values could only be calculated for the parsimony gene tree collection because of computational limits (100 maximum likelihood searches on 557 data sets was prohibitive). Support was >95% for all nodes in the species tree derived from the parsimony gene trees except for the rosid clade, which was only supported at 48% (Fig. 3). Bootstrap II support values were also moderate for the Rosid clade in both parsimony and likelihood gene tree analyses (71% and 68% respectively). In addition, the relationship within the legumes, which conflicts between the two optimal species trees (*Glycine *+ *Lotus *versus *Medicago *+ *Lotus*), is weakly supported (66%) in the likelihood analysis, but strongly supported (99%) in the parsimony analysis.

Because our analyses supported two different trees depending on which collection of gene trees was used, we examined whether these two trees were statistically distinguishable on the basis of the data at hand. Let *T*_*L *_be the optimal species tree found based on the likelihood gene trees (identical to the accepted tree) and *T*_*P *_be the optimal tree found with the parsimony gene trees. We examined the difference in support for these two trees based on either the likelihood gene tree collection or the parsimony gene tree collection using the analog of the paired-sites test described in the Methods. Based on the parsimony gene trees, there was weak but significant support (*P *= 0.04) for a difference between *T*_*L *_and *T*_*P*_. Of the 577 gene trees, 403 showed no difference in unrooted duplication scores between the two trees; 101 had better (lower) scores for *T*_*P *_compared to *T*_*L*_; 77 had better scores for *T*_*L*_. On the other hand, there was no significant difference in support (*P *= 0.36) based on the likelihood gene tree collection for a difference between *T*_*L *_and *T*_*P*_. Of the 577 gene trees, 408 showed no difference in unrooted duplication scores between the two trees; 85 had better (lower) scores for *T*_*P *_compared to *T*_*L*_; 84 had better scores for *T*_*L*_. These results are congruent with the bootstrap II comparisons in that they suggest the parsimony gene tree collection makes a more decisive claim about the difference in the species trees than does the likelihood gene tree collection.

## Discussion

### Phylogenetic sparseness of the EST data

Phylogenomic data sets, whether derived from whole genome sequencing [15], database mining [18], or EST assemblies [20,27] have yet to combine into one analysis more than a few hundred clusters of sequence homologs ("loci"). The reasons for this are many, but a primary one is the tradeoff between completeness of a data set and lack of homology that eventually limits cluster construction. Sanderson and Driskell [50] and Driskell et al. [18] illustrated this graphically by showing the low density of concatenated data matrices assembled from GenBank data mining approaches. *Density *can be defined as the fraction of sequences present in a "data availability matrix" consisting of all taxa in an analysis by all clusters. The reason why phylogenetic data matrices derived from whole genome analyses do not include *all *the genes in the genome is partly because lack of homology between sequences in these taxa limits how many taxa actually share the gene in common (either due to gene loss or excessive divergence). Clusters used in phylogenetic analysis are sometimes explicitly constructed to have all taxa or a minimum fraction of such taxa [51], thus keeping data density above a threshold, but also greatly limiting the eventual size of the data matrix.

EST-based studies also seem to fit into this same paradigm. For example, using small EST libraries to identify orthologous clusters of ESTs, Hughes et al. [20] constructed supermatrices with 71% missing data, and this was *after *exclusion of most of the data because of extensive paralogy. In our data, for the cluster set used for most analyses, we identified 88,864 clusters for the seven taxa. However, 79,122 of these were singleton clusters, meaning that a whopping 75% of the original 105,453 TCs did not pass our minimal homology threshold. Moreover, as only 577 of the remaining clusters were actually potentially *phylogenetically *informative, the final density in the phylogenetic data availability matrix could not possibly exceed 577/88,864 or 0.6%. Higher densities are possible if the cluster assembly stringency is relaxed, but as we have seen, this leads to very heterogeneous clusters with few regions of homology – presumably engendering downstream problems in subsequent phylogenetic analysis.

This very small fraction of the EST data that appear to be potentially useful for phylogenetic studies raises questions about the relative costs and benefits of obtaining EST data for phylogenetic work [20]. However, several other factors are important to consider. First, using available EST libraries as tools to screen for loci useful for phylogenetic inference may justify their expense in a small number of pilot taxa. Primers can be developed for later use in extensive taxon surveys (e.g. [52]). Second, as local alignment tools (e.g. [53]) and phylogenetic inference algorithms improve, it should be possible to assemble clusters with more heterogeneity and distant homologies, and hence exploit more of the original data. Finally, it may be necessary to view the problem as one that will eventually be overcome by improvements in technology and reductions in expense. After all, for many single loci sequenced in conventional phylogenetic analysis, most sites are conserved and uninformative. The only factor that makes this palatable is the (now) relative inexpensiveness of sequencing technology.

### Extent of duplication and implications for species tree inference

Among the 577 phylogenetically informative clusters, most showed evidence of gene duplication by conflicting with the accepted species tree. Even if we conservatively regard inparalogs as multiple alleles or multiple accessions of the same locus, there are 351 clusters that show at least one out-duplication when reconciled against the species tree using the likelihood gene trees. If we take a more liberal view, counting all duplications, then 535 of the clusters show evidence of duplication. Similar numbers obtain if the parsimony gene trees are used. Since duplications are minimized across all rootings of the gene trees, our estimated number of duplicated loci is probably somewhat lower even than the true value. On the other hand, the fact that the gene trees themselves have error is unaccounted for by our methods, and, failing to take uncertainty into account may inflate the inferred number of duplications [13]. Regardless of these considerations, the fraction of the phylogenetically useful data in clusters that are locked up in gene families in plants, as opposed to single-copy genes, seems to be extremely high. To exploit the nuclear genome in plants to build species trees therefore seems to require methods that can handle extensive duplication (and gene loss or failure to sample), such as GTP or alternative frameworks [11,54].

### Performance of species tree inference

Despite the extensive heterogeneity in the data themselves, and the complex informatics pipeline that ultimately filtered out most of the original data, remarkably strong signal for the accepted species phylogeny was evident in the GTP analysis. The GTP analysis of the likelihood gene trees yielded the correct "accepted" species tree. The analysis of the parsimony gene trees yielded a species tree close to the accepted tree (which was ranked fourth out of 945). We find these results both surprising and promising for three reasons. First, gene families are subject to a variety of processes that can destroy the hierarchical signature of phylogenetic history, such as gene conversion between paralogs. Methods are available to detect such events [55,56] and to incorporate them into phylogenetic inference [57], but the latter are still in their infancy.

Second, the EST data themselves were "messy" compared to other data sets we have examined [18]. EST tentative consensus sequences, which formed the start of our analysis, are themselves assembled from individual short EST sequences using complex and assumption-laden informatics protocols [30]. Among the factors that these assemblies contend with are filtering contaminants, correct assembly of pieces of the same paralog in gene families, and handling of alternative splicing, all in the presence of the usual issues raised in local and global homology algorithms. Though EST data have been widely used in evolutionary studies (e.g. of whole genome duplications: [48]), they have rarely been used en masse in phylogenetic analysis of any taxon [20,51], and it was reasonable to think that one cause for this was that these complexities overcame any underlying signal. Apparently this is not the case.

Finally, GTP has only been used to build species trees in a few studies. Although a few issues have been raised in criticism of GTP (see below), one cannot help but think that GTP has not been used more either because of lack of software tools, or lack of data. Although many implementations of gene tree reconciliation are available [13,40,54], few tools for GTP itself have been available except for Page's COMPONENT [38] and later GeneTree programs [39]. Neither of these is set up to handle large numbers of loci easily or is scriptable, a necessity for much high throughput informatics work. Moreover, the search strategies rely only on branch swapping from random starting trees. If GTP is as difficult an optimization problem as maximum parsimony, with as messy data, experience suggests that this heuristic is not likely to perform very well. However, we have not solved that particular problem either. Instead, we *avoided *it by implementing an exact exhaustive enumeration possible only because of the small species tree in our problem.

Another reason for the lack of GTP studies may be the lack of available gene family data for many taxa. Phylogeneticists have done their best to filter out gene families in the search for single-copy "magic bullets" that are easily sequenced by direct PCR, and to avoid cloning, Southern blots, or other labor-intensive techniques that are often necessary to initially identify paralogous copies of loci [58]. However, large databases of protein families exist and have been relatively underexploited for species tree inference (except see Cotton and Page's [12] analysis of the HOVERGEN database). For many taxa that phylogeneticists find interesting, however, such data are simply not available. The taxa are not model species by and large, and there has been no compelling reason to seek a diversity of loci in relatively obscure taxa. This will probably change as more and more sequencing projects and EST libraries build bridges to nonmodel taxa.

Criticisms of GTP are numerous [59], and many reflect the same concerns as have been raised about supertree analysis [60] – in particular, that by taking a set of trees as the input, information about the uncertainty in those trees is lost (and hidden information within each data set cannot synergistically emerge). Certainly some number of duplications are inferred incorrectly simply because the gene tree is wrong [13]. To address this, relative clade support scores can be incorporated when reconciling gene trees with species trees [13]. However, the sheer volume of gene trees used here apparently overcame the errors associated with any one incorrect gene tree, implying a lack of systematic bias in the gene tree estimates, at least when likelihood was used to infer the gene trees. A more niggling issue is that the standard GTP algorithms all still require binary input trees. Chang [61] has developed an algorithm that solves this problem, but it is not yet implemented. Both of these problems can be addressed at least partly through bootstrap procedures [12], which, when constrained to generate binary gene trees, sample across much of the diversity that is entailed by multifurcations arising from either lack of data for that node or conflicting signals.

### Future work

Currently the main factor limiting the application of GTP to species tree inference seems to be a paucity of implemented tree search heuristics. Three related algorithmic challenges remain in this arena. First, gene tree uncertainty has to be integrated more directly into the tree reconciliation calculations. Durand et al. [13] developed algorithms to calculate improved duplication scores in the presence of gene tree uncertainty and demonstrated the dramatic reduction in estimated score that can ensue. These or similar approaches must ultimately be imbedded in GTP algorithms. Second, multifurcations in the gene tree and species tree have to be accommodated [61]. Finally, to address the growing size of data sets, it will be necessary to integrate these aspects of the GTP problem with whatever tree search heuristics are developed, so that redundant re-calculation of scores for subtrees are avoided.

On the data analysis side, the size and taxonomic diversity of EST libraries will continue to grow, and our results suggest that these will be useful sources of data in the future for inferences about phylogeny. However, much work remains on the pre-processing side of the analysis, prior to gene tree construction and GTP analysis. The assembly of ESTs is a computationally and biologically challenging problem, especially in light of the high frequency of duplication in plant genomes, and the not infrequent occurrence of alternative splicing [30]. Perhaps the greatest challenge will be to develop methods that properly account for ascertainment bias: the failure to sample all paralogs in a gene family for some or all taxa. Although model-based approaches (e.g. [54]) to gene tree reconciliation offer a direct route to incorporate models of sample bias into the problem, these are computationally expensive methods, and it may be possible to use faster weighting schemes in some modification of the GTP framework.

Finally, the ubiquity of whole genome duplications (e.g. [5,62]) has important implications for inferring species trees from gene families. Page and Cotton [63] looked for clustering of episodes of duplication in vertebrate gene families but found little evidence for it based solely on the phylogenetic position of the duplications. Subsequently [64] they added to their phylogenetic approach inferred duplication times and then found support for an ancient round of accelerated duplication rates in vertebrates, though not the recent episode that has been reported elsewhere [65]. Their approach complements a much more widely used approach of examining the distribution of duplications ages for peaks at different points in time (e.g. [66]). In addition to providing insights into genome evolution, these approaches suggest that supplementing the GTP inference problem with divergence time information to constrain its structure may be profitable, if only the accuracy of such information can be assured.

## Methods

### Sequence data and gene trees

We downloaded EST data for seven plant taxa, including six angiosperms (*Oryza sativa*, *Solanum tuberosum*, *Arabidopsis thaliana*, *Glycine max*, *Lotus japonicus*, *Medicago truncatula*) and one conifer, *Pinus *(Fig. 2) to serve as an outgroup. Data were obtained from the TIGR Gene Indices Database [67,68] (Table 1). Initial data analysis protocols were similar to those reported in [48]. We extracted all TCs (tentative consensus sequences) for each taxon and used the EMBOSS program getorf [69] to find open reading frames of at least 500 nt in length in the sense direction. Default settings were used (ORF defined as a region between stop codons according to the standard genetic code). These filtered TCs were then used in subsequent analyses.

Clusters of homologous TCs were obtained using all-by-all BLAST nucleotide similarity searches [70] on the filtered and trimmed TCs (low-complexity filter DUST turned on; maximum Expect (E) value of 1.0e-10). BLAST was undertaken on nucleotide sequences despite the high level of divergence at third codon positions because of the possibility of mistaken amino acid translations based on incorrect ORF identifications in these data in which alternative splicing was not uncommon. Single-linkage clustering was used to assemble clusters based on BLAST output (program *blink *available at MJS's web site [71]; additional utility scripts available from authors). High levels of within-cluster heterogeneity among sequences can lead to severe alignment problems [72]. Therefore, a pair of sequences was considered as a hit if it was reported as a BLAST hit and it surpassed a minimum "hit fraction" of 0.70 for each sequence, i.e., at least 70% of each sequence must align to the other sequence with E values lower than the threshold – though not necessarily in a single contiguous hit. The threshold was imposed symmetrically for both query and target sequence. The value 0.70 was experimentally determined by simultaneously attempting to maximize the number of sequences assigned to clusters and minimizing the heterogeneity, both in terms of sequence divergence and length differences, of the resulting clusters.

Resulting clusters were screened for potential phylogenetic informativeness. To provide potential information in a GTP analysis, which fundamentally requires a rooted species tree and one or more rooted gene trees, the gene trees and the clusters used to construct them must consist of three or more sequences from three or more species. However, because we are not using external evidence to root the gene trees but rather are examining all duplication scores across all possible rootings, our gene trees must have at least four sequences. If a gene tree with three sequences only is rerooted, it will be congruent with all rooted species tree for *some *gene tree rooting, and therefore it will not provide any information to discriminate among species relationships in the GTP analysis. If on the other hand, the gene trees were rooted using a molecular clock or midpoint rooting, for example, then clusters with only three sequences could potentially incur duplication scores that differed from species tree to species tree.

Once the clusters were screened for informativeness (with specific regard to gene tree parsimony), we used the global alignment program Clustal W [73] to align nucleotide sequences within the clusters. A sample of alignments was checked manually for obvious alignment mistakes, none were found, and consequently the alignments were not edited further. Gene trees were reconstructed for each cluster using heuristic maximum parsimony and maximum likelihood implemented in PAUP* 4.0b10 [74]. Because only binary trees may be used in available algorithms for gene tree parsimony, zero-length branches were not collapsed in either method. Heuristic parsimony searches consisted of simple-addition sequences with tree-bisection-reconnection branch swapping, keeping a maximum of 10000 equally parsimonious trees (which was never exceeded). Heuristic maximum likelihood searches used a neighbor-joining starting tree followed by TBR branch swapping, time-limited to 6 hours, using an HKY85 + Γ model of evolution in which all parameters were estimated from the data. All phylogenetic analyses were conducted on a dual Xeon 2.80 Ghz CPU with 3 GB of RAM or on a 35 node Linux cluster, in which the head node is a dual Xeon 2.66 ghz CPU with 3 GB RAM and each node is a dual AMD 1.4 Ghz CPU with 1 GB RAM.

To construct a confidence set of trees for each cluster in parsimony analyses, we bootstrapped the sequence data (100 pseudoreplicates, saving each gene tree, or set of trees, each weighted by the inverse of the number of trees found for that particular replicate). Searches were conducted with the same settings as for searches on the original clusters. The computational overhead was too high to do the same for maximum likelihood (worst case running time: six hours × 100 replications × 557 data sets).

### Gene tree reconciliation: duplication scores on the accepted species tree

To reconcile the gene trees to species trees by minimizing the number of duplication events, we implemented the algorithm of Zmasek and Eddy [32] in a C program available from MJS at his web site [71]. This algorithm runs, under the rarely expected worst case, in *O*(*n*^2^) time [32], but its average behavior is much better, as shown both by Zmasek and Eddy's experimental results [32] and our experience with the present data set. We implemented their algorithm in C to run quickly for the large numbers of gene trees and species trees analyzed in this paper: each gene tree parsimony analysis had to reconcile 557 gene trees under all possible rootings for each of 945 species trees. Although Durand et al.'s [13] recently released NOTUNG 2.1 program is quite full featured and would have been an appropriate tool for this task, its Java implementation and requirement that it be re-executed for each species tree/gene tree pair made it too slow for this problem.

Other criteria can be used to reconcile gene trees to species trees such as the sum of duplications plus losses or, for recently diverged lineages, coalescent depth [75]. We chose not to incorporate the number of losses into the optimality criterion because the data sets for the current study, largely derived from ESTs, are exceptionally prone to incomplete sampling, and a true evolutionary loss is therefore difficult to distinguish from mere ascertainment bias. Moreover, adding losses to the optimality criterion introduces the difficult problem of weighting the relative importance of duplications, losses due to evolutionary deletion, and "losses" due to sampling omissions. The duplication score alone is expected to be a more robust indicator of gene family diversity in these circumstances [12].

Reconciliation of a gene tree to a species tree requires that both trees be rooted [8]. The species tree of angiosperms is rooted with an outgroup to angiosperms among seed plants, the conifer, *Pinus*. However, outgroup rooting is not possible for the gene trees, because, for example, a gene tree might have two paralogs from *Pinus *in different parts of the tree, leaving the position of the root uncertain. Occasionally, the root may be inferred in simple scenarios in which a single duplication has occurred prior to all taxa in the analysis and the root is clearly between the two paralog trees, but in general, this will not be the case. Therefore, as suggested previously [13,32], we reconciled the species and gene tree by evaluating the duplication score for all possible roots of the gene tree, selecting the root(s) that minimize the number of duplications inferred. Some EST clusters produced multiple equally parsimonious trees. In these cases an average duplication score was constructed across the set of equally parsimonious trees.

Because clusters lacking duplications are of special significance to species level phylogenetics, e.g. they can potentially be concatenated in "supermatrix" analyses, we estimated their occurrence in the data. A cluster was scored as lacking duplications if *all *equally parsimonious trees for that cluster had an unrooted duplication score of zero. These values are reported both for all duplications and for only out-duplications.

### Gene tree parsimony: finding the optimal species tree

Because of the relatively small size of the species tree, gene tree parsimony searches for the optimal species tree were implemented by exhaustively enumerating all 945 species trees (rooted with *Pinus*), and calculating the summed gene duplication scores across all gene trees for each of these species trees. This procedure was repeated for both parsimony and likelihood collections of gene trees. This strategy obviously would not be feasible for species trees much larger than this. A benefit of exhaustive enumeration is that it provides the exact distribution of GTP scores across all the species trees. This allowed, among other things, a ranking of all species trees according to GTP score and a comparison of the relative position of the optimal GTP tree and the true tree.

### Support levels and hypothesis tests

Little work has been done to develop confidence assessments in GTP analyses, per se, although several authors have taken a bootstrap approach to identification of orthologs with gene tree reconciliation [32]. Cotton and Page [12] suggested a bootstrap analysis to account for gene tree uncertainty, in which each of *k *data sets used to generate the *k *gene trees is bootstrapped *N *times, generating a set of *k *bootstrap profiles. Then a higher level GTP bootstrap analysis is done by taking the *i*th tree from each of the *k *profiles and performing a complete GTP search for the species tree, generating species tree *i*, and repeating this for *i *= 1, ..., *N*. The collection of *N *species tree then forms a confidence set of species trees, and majority rule consensus is used to summarize support, as in conventional bootstrapping [76]. We refer to this as *Bootstrap I*.

An alternative bootstrap procedure uses the gene trees themselves as the sampling unit. In a single bootstrap replicate, a set of *k *gene trees is assembled by sampling from the original set of *k *gene trees randomly with replacement. Then a species tree is built by GTP, and the process is repeated *N *times. Again a majority rule tree can be constructed. We refer to this as *Bootstrap II*.

Finally, because the optimal species tree may be different from the accepted species tree in Figure 2, it is useful to test whether there is a significant difference in support from the gene duplication data. For this, we propose a simple analog to paired sites tests used extensively for parsimony and likelihood tree inference (reviewed in [77]). For each gene tree, we calculate the duplication score on tree 1 and tree 2. Under the null hypothesis of equal support for the two trees, the mean difference in score of these across sites should be zero. A paired *t*-test provides a test of significance taking the variance into account. As is now well known however, if one of the two trees is the optimal tree (as it will be here), the test is one-sided and the *P*-value must be the appropriate one-sided version [77]. Additional analogous tests presumably could be constructed to account for multiple test issues that might arise if we examined many trees [78].

## Authors' contributions

MJS wrote the code to implement GTP. MMM performed a majority of the informatics data analyses. The authors participated equally in writing the manuscript.

## Figures and Tables

**Table 1:** Sequence and cluster data for each taxon

Taxon^a^	Release^b^	Original TCs^c^	MaxORFs^d^	Clusters^e^	Final TCs^f^
*Arabidopsis thaliana*	12.1	28900	23737	343	729
*Glycine max*	12.0	31928	13930	538	1065
*Lotus japonicus*	3.0	12485	3116	365	452
*Medicago truncatula*	8.0	18612	12254	528	852
*Oryza sativa*	16.0	36381	25842	199	418
*Pinus*^g^	6.0	23531	13949	159	315
*Solanum tuberosum*	10.0	21063	12625	378	705
Total		172900	105453	577	4536

^a ^Taxon as given by TIGR for the EST collection assembled in the Gene Index Database.

^b ^Versions used in this paper, current as of 18 February 2006.

^c ^The 363,971 sequences in the database for these taxa were screened to include only those sequences assembled by TIGR into Tentative Consensus (TC) sequences.

^d ^TCs were trimmed to the largest sense-direction ORF that was at least 500 nt in length; shorter sequences were discarded.

^e^Number of clusters in which the taxon is represented, after screening for phylogenetic informativeness (at least three taxa and at least four sequences).

^f ^Total number of sequences from each taxon in the final set of clusters.

^g^TIGR assembled this library from several species of *Pinus*.

**Table 2:** Effects of hit fraction threshold on cluster assembly. Bold indicates the threshold chosen for the current study.

Hit fraction^a^	Clusters^b^	Singletons^c^	Phylogenetically informative clusters^d^	Max size^e^	TCs in phylogenetically informative clusters^f^
0.0	39924	26782	4423	6565	54051
0.1	47798	32824	4079	1947	42406
0.2	57229	41327	3324	1362	29403
0.3	64691	48864	2561	330	21504
0.4	71333	56383	1876	117	15457
0.5	77564	63890	1340	98	10721
0.6	83435	71539	897	95	7105
**0.7**	**88864**	**79122**	**577**	**94**	**4536**
0.8	94296	87186	324	92	2529
0.9	99843	95975	103	89	872
1.0	105144	104860	1	6	6

^a ^Minimum proportion of sequence similarity based on BLAST's pairwise comparisons. The hit fraction determines whether a sequence is linked to another (if a pair is linked, they will be placed in the same cluster) and thus affects the level of heterogeneity within clusters and the number of assembled clusters. Original number of sequences is 105,453 TCs.

^b ^Total number of assembled clusters.

^c ^Number of single-sequence clusters.

^d ^Phylogenetically informative clusters for this study are those that include at least three species and at least four sequences.

^e ^Number of tentative consensus sequences (TCs) in the largest phylogenetically informative cluster.

^f ^Total TCs in all phylogenetically informative clusters.

**Table 3:** Distributions of cluster sizes by number of taxa

Number of taxa in cluster	Number of clusters
1	86022
2	1986
3	478
4	162
5	90
6	67
7	59

**Table 4:** Distributions of cluster sizes by number of tentative consensus sequences (TCs)

Number of TCs in cluster	Number of clusters
1	79122
2–3	8645
4–9	930
10–94	167

**Table 5:** Distribution of duplication scores among clusters

	MP gene trees	ML gene trees
Number of clusters with zero duplications	40	42
Number of clusters with zero *out*-duplications	211	226
Maximum duplications in any cluster	83	81
Maximum *out*-duplications in any cluster	20	17
